# Beyond Credentials: Understanding Educational Gradients in Cognitive Function at Midlife

**DOI:** 10.1002/alz.088049

**Published:** 2025-01-09

**Authors:** Eric Grodsky, John Robert Warren, Chandra Muller, Jennifer J. Manly, Adam M. Brickman, HSB

**Affiliations:** ^1^ University of Wisconsin ‐ Madison, Madison, WI USA; ^2^ University of Minnesota, Minneapolis, MN USA; ^3^ University of Texas at Austin, Austin, TX USA; ^4^ Department of Neurology, Columbia University, New York, NY USA; ^5^ Columbia University Irving Medical Center, New York, NY USA

## Abstract

**Background:**

A substantial body of research points to the persistence of educational credential gradients in cognitive function across the life course, from early adulthood through old age. Fewer studies have considered the roles of *educational opportunities* and *achievements* early in the life course in shaping (1) educational credential gradients or (2) variation in cognitive function among those with the same credentials. As a result, researchers underestimate the contribution of education writ large to variation in cognitive performance later in life.

**Method:**

We analyze data from the nationally representative High School and Beyond (HSB) cohort, which has followed ∼25,500 people from high school in 1980 through age ∼60 in 2021‐22. The 2021‐22 round of HSB gathered high quality measures of cognition across multiple domains; the 1980‐1992 rounds gathered extensive information about educational opportunities, achievements, and attainments.

**Result:**

We demonstrate that high school educational opportunities, and especially academic achievements, account for most of the credential gradient in cognition on selected cognition measures among adults who attended high school in the U.S. in 1980 as they approach 60 years of age. High school educational opportunities and academic achievements also help account for some of the variation in cognition among those with the same educational credential.

**Conclusion:**

Educational credentials commonly employed to understand the contribution of education to cognitive aging therefore risk both mischaracterizing and understating the contributions of education to cognitive function and change over the life course.

